# Impact of Glutathione and Vitamin B-6 in Cirrhosis Patients: A Randomized Controlled Trial and Follow-Up Study

**DOI:** 10.3390/nu12071978

**Published:** 2020-07-03

**Authors:** Chia-Yu Lai, Shao-Bin Cheng, Teng-Yu Lee, Yung-Fang Hsiao, Hsiao-Tien Liu, Yi-Chia Huang

**Affiliations:** 1 Division of General Surgery, Department of Surgery, Taichung Veterans General Hospital, Taichung 40705, Taiwan; chiayulai@vghtc.gov.tw (C.-Y.L.); sbc@vghtc.gov.tw (S.-B.C.); langhsky1981@gmail.com (H.-T.L.); 2 Graduate Program in Nutrition, Department of Nutrition, Chung Shan Medical University, Taichung 40201, Taiwan; summerbreathsrelax@gmail.com; 3 School of Medicine, Chung Shan Medical University, Taichung 40201, Taiwan; tylee@vghtc.gov.tw; 4 Division of Gastroenterology and Hepatology, Taichung Veterans General Hospital, Taichung 40705, Taiwan; 5 Department of Nutrition, Chung Shan Medical University, Taichung 40201, Taiwan; 6 Department of Nutrition, Chung Shan Medical University Hospital, Taichung 40201, Taiwan

**Keywords:** Vitamin B-6, glutathione, oxidative stress, Child–Turcotte–Pugh score, liver cirrhosis

## Abstract

Vitamin B-6 and glutathione (GSH) are antioxidant nutrients, and inadequate vitamin B-6 may indirectly limit glutathione synthesis and further affect the antioxidant capacities. Since liver cirrhosis is often associated with increased oxidative stress and decreased antioxidant capacities, we conducted a double-blind randomized controlled trial to assess the antioxidative effect of vitamin B-6, GSH, or vitamin B-6/GSH combined supplementation in cirrhotic patients. We followed patients after the end of supplementation to evaluate the association of vitamin B-6 and GSH with disease severity. In total, 61 liver cirrhosis patients were randomly assigned to placebo, vitamin B-6 (50 mg pyridoxine/d), GSH (500 mg/d), or B-6 + GSH groups for 12 weeks. After the end of supplementation, the condition of patient’s disease severity was followed until the end of the study. Neither vitamin B-6 nor GSH supplementation had significant effects on indicators of oxidative stress and antioxidant capacities. The median follow-up time was 984 d, and 21 patients were lost to follow-up. High levels of GSH, a high GSH/oxidized GSH ratio, and high GSH-St activity at baseline (Week 0) had a significant effect on low Child–Turcotte–Pugh scores at Week 0, the end of supplementation (Week 12), and the end of follow-up in all patients after adjusting for potential confounders. Although the decreased GSH and its related enzyme activity were associated with the severity of liver cirrhosis, vitamin B-6 and GSH supplementation had no significant effect on reducing oxidative stress and increasing antioxidant capacities.

## 1. Introduction

The progression of liver cirrhosis is a multifactorial process that involves repetitive hepatocyte damage and subsequent irreversible scarring. In addition, increased oxidative stresses and decreased antioxidant capacities have been observed in cirrhotic patients [1,2,3,4,5]. Therefore, it is important for cirrhotic patients to restore the balance of oxidative stress and antioxidant capacities or even lean to a stronger antioxidant defense capacity to protect the liver from further damage, as well as decelerate the disease progression.

Glutathione (GSH) and its related enzymes [i.e., glutathione *S*-transferase (GSH-St), glutathione peroxidase (GSH-Px), glutathione reductase (GSH-Rd)] are well known antioxidants of human body that capture toxic electrophilic xenobiotics and scavenge free radicals [6]. GSH is mainly synthesized by cysteine, glycine, and glutamate in the liver, and is oxidized to glutathione disulfide (GSSG) via GSH-Px, and is reduced back to GSH by GSH-Rd. Since the liver is the mainstay of total body GSH turnover and accounts for greater than 90% of GSH inflow into the systemic circulation [7], the endogenous GSH basal appearance rate in cirrhotic patients have been reported to have a 50% reduction compared to healthy subjects, and the GSH appearance rate is significantly correlated with liver function [8]. Leach et al. observed that patients with non-alcoholic fatty liver disease had significantly lower levels of GSH and GSH-Px activity when compared to healthy controls [9]. Liver dysfunction appears to impair GSH synthesis, and the increment of GSH status might be effective in maintaining the GSH level and its related antioxidant capacities for patients with liver cirrhosis. Although four weeks supplementation of oral GSH (1000 mg/d) neither changes the erythrocyte GSH concentration nor reduces the level of oxidative stress indicators in healthy adults [10], the plasma GSH concentration was shown to be significantly increased after three and six months of oral GSH supplementation (1000 mg/d) in healthy subjects [11]. The GSH supplemented period appears to be crucial for effectiveness and a minimum of three months is necessary for healthy subjects. However, no oral GSH supplementation has been implicated in liver cirrhosis patients, and the efficacy of oral GSH supplementation on oxidative stress and antioxidant capacities has not yet been determined.

As the rate-limiting substrate for GSH biosynthesis, cysteine can either be obtained from the diet or through the transsulfuration pathway for the conversion of homocysteine to cysteine via pyridoxal 5′-phsophate (PLP, the physiological coenzyme form of vitamin B-6) acting as an essential coenzyme [12]. Since the liver is the main site for the storage and metabolism of vitamin B-6, deficient vitamin B-6 may indirectly interfere with GSH synthesis, and subsequently affect the antioxidant capacities of GSH and its related enzymes. A previous animal study indicated that inadequate vitamin B-6 caused a reduction in the GSH level, as well as the activities of GSH-Px and GSH-Rd in the liver tissue of rats [13]. Although a single dose of vitamin B-6 supplementation (50 mg/d) did not show a significant effect on the GSH and GSSG levels and activities of GSH-Px, GSH-Rd, and GSH-St in patients with hepatocellular carcinoma [14], the combination of vitamin B-6 and GSH supplementation are likely to have antioxidative effects in liver cirrhosis patients. Since an impaired vitamin B-6 and GSH status has been observed in patients with liver cirrhosis [15,16,17,18,19], a strategy is required to either maintain adequate or increase vitamin B-6 and GSH status in order to cope with the altered oxidative stress and antioxidant capacities in this specific group of patient. However, it remains unclear whether supplementation with vitamin B-6 or GSH could compensate for their reduced status, and improve the antioxidant capacity of liver cirrhosis patients. This study aimed to evaluate whether a single or combination of vitamin B-6 and GSH supplementation had effects on oxidative stress and antioxidant capacities in patients with liver cirrhosis. In addition, we followed patients after the end of supplementation to evaluate the association of vitamin B-6 and GSH with disease severity.

## 2. Subjects and Methods

### 2.1. Study Design and Sample Size Calculation

This was a double-blind randomized clinical trial (clinical trial no. NCT02321579, ClinicalTrials.gov Protocol and Results Registration System) with a 2 × 2 factorial design, and a follow-up study. Based on the result of Richie et al. [11] that indicated that the blood total GSH level increased from 1.04 ± 0.11 mol/mL to 1.22 ± 0.31 mol/mL in healthy adults after 6 months of 250 mg/d oral GSH supplementation, we then assumed there would be a difference of 17% in the mean plasma GSH level between the placebo and GSH supplemented group, with a standard deviation of 0.2 mol/mL. The minimal sample size required was 12 patients per group at a power of 80% and a two-sided test with α = 0.05.

### 2.2. Subjects

We recruited study patients from the Division of General Surgery and Division of Gastroenterology and Hepatology of Taichung Veterans General Hospital, Taiwan. Patients were diagnosed with liver cirrhosis by clinical presentation, as well as histopathological, biochemical, and radiologic reports. The Child–Turcotte–Pugh classification scheme [20] was utilized as the severity score of liver cirrhosis according to serum albumin and total bilirubin concentrations, international normalized ratio (INR), and the degree of ascites and hepatic encephalopathy; a score 5–6 is classified as Class A, score 7–9 is Class B, and score 10–15 is Class C. Patients were excluded if they were <20 or >80 years, had decompensated cirrhosis in a critical clinical condition, pregnant or lactating, receiving chemotherapy, or if they had diabetic, cardiovascular, renal or chronic inflammatory diseases. This study was approved by the Institutional Review Board of Taichung Veterans General Hospital (IRB TCVGH No. SF14261B), and all patients signed the informed consent prior to participation in the study.

### 2.3. Intervention and Follow-Up Procedure

None of the study patients received vitamin B-6 or GSH supplementation in the month prior to the study. In total, 61 patients were randomly assigned to one of four study groups using the next sequential number from the randomization list generated by QuickCalus (GraphPad software Inc., San Diego, CA, USA). The four study groups consisted of a placebo group (dextrin starch, New Health Entereprise, Inc., Irvine, CA, USA, *n* = 14), vitamin B-6 group (50 mg/d pyridoxine-HCl, Nature’s Way Products, Inc., USA, *n* = 14), GSH group (500 mg/d L-glutathione, Chambio Co., Ltd., Taichung, Taiwan, *n* = 18), and a B-6 + GSH group (50 mg/d vitamin B-6 and 500 mg/d GSH, *n* = 15).

Supplementation was administered for 12 weeks, and the tablets were indistinguishable in terms of color, size, and shape. Patients were instructed to take tablets daily, and ensure that they were maintaining their usual dietary intakes and daily activities during the intervention period. To monitor the compliance, when patients returned to the clinic to pick up additional supplements, any unused tablets from the previous 4 weeks were returned and counted.

**Figure 1:**
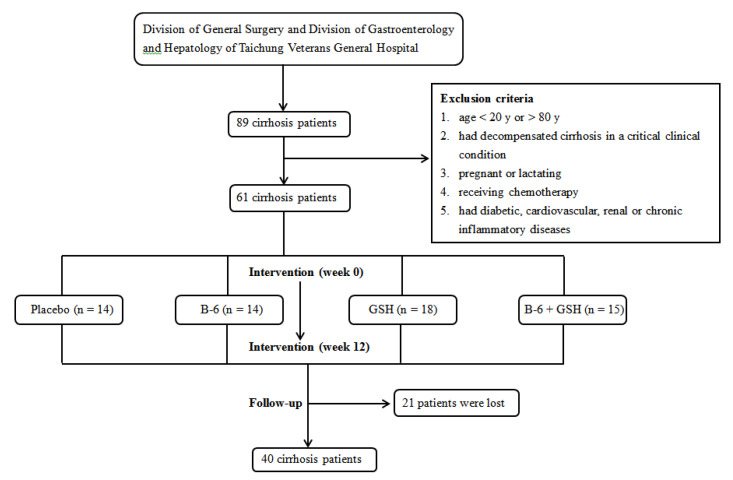
The design and flow diagram of the study.

After the end of intervention (Week 12), we followed up 61 patients from the date that the intervention was completed to the end of the study on 31 October 2019. During the follow-up period, patients did not take either vitamin B-6 or GSH supplementation. The primary outcome was disease severity (Child–Turcotte–Pugh score). The design and flow diagram of the study is shown in Figure 1.

### 2.4. Data Collection and Biochemical Measurements

Age, sex, smoking, and drinking habits were recorded, and height, weight, systolic and diastolic blood pressures after a resting period of at least 5 min were measured at baseline (Week 0), and body mass index (BMI, kg/m^2^) was calculated.

Fasting blood samples were drawn and collected in vacutainer tubes (Becton Dickinson, Rutherford, NJ, USA) either with or without anticoagulant at Week 0 and Week 12. Serum or plasma was immediately separated after blood was drawn by speed centrifugation at 2500 rpm, 4 °C, for 15 min. Samples were either directly analyzed or frozen stored at −80 °C until analysis. Serum samples were used to measure albumin, total bilirubin, INR, alanine aminotransferase (ALT), aspartate aminotransferase (AST), and creatinine using an automated biochemical analyzer. Plasma samples were used to determine levels of malondialdehyde (MDA), cysteine, PLP, GSH, GSSG, and trolox equivalent antioxidant capacity (TEAC), as well as activities of GSH-Px, GSH-Rd and GSH-St. As an oxidative stress indicator, MDA levels were measured with thiobarbituric acid reactive substances using a fluorescence spectrophotometer [21]. Cysteine and PLP were quantified by high performance liquid chromatography with a fluorescence detector based on modified methods [22,23]. The levels of GSH, GSSG, and the activities of GSH-Px, GSH-St, SOD and catalase were determined using respective commercial kits (BioVision Incorporate, Milpitas, CA, USA; Cayman Chemical Company, Ann Arbor, MI, USA), while GSH-Rd activity was assessed according to the method of Carlberg and Mannervik [24]. TEAC was assessed according to a previously described method [25].

### 2.5. Statistical Analysis

Statistical analysis was performed using SAS statistical software package (version 9.4; Statistical Analysis System Institute Inc., Cary, NC, USA). Intention-to-treat analysis was applied in this clinical trial. The Shapiro–Wilk test was used for the normality test of sample distributions. Demographic, clinical, and biochemical parameters were compared using a one-way analysis of variance or Kruskal–Wallis one-way analysis of variance of ranks to determine significant differences among groups at Week 0 and Week 12. Chi-square or Fisher’s exact tests were used in the analyses of categorical variables. Paired *t*-test or Wilcoxon signed rank test were used to compare the difference in biochemical parameters within groups between Week 0 and Week 12. Multiple linear regression was used to assess the effect of supplementation (placebo = 1, B-6 = 2, GSH = 3, B-6 + GSH = 4) on oxidative stress and antioxidant capacities after adjusting for age, sex, BMI, baseline (Week 0) Child–Turcotte–Pugh score, and smoking and drinking habits. In addition, multiple linear regressions with Child–Turcotte–Pugh score at Week 0, Week 12, and the end of follow-up as the dependent variable was performed to assess the effect of oxidative stress and antioxidant capacities on the severity of liver cirrhosis after adjusting for potential confounders. Results were considered statistically significant at two-tailed, *p* < 0.05.

## 3. Results

No patients dropped out during the intervention period. Overall, there was 95.02 ± 1.18% and 92.62 ± 1.67% (mean ± standard error of mean) compliance in all patients by tablet counts at Week 0 and Week 12, respectively, and the compliance did not significantly differ among groups. In addition, no serious adverse effects were reported by the study patients during the intervention period.

Table 1 lists the demographic and clinical characteristics of the patients at Week 0 and Week 12. Study patients were in Class A and B of the Child–Pugh score, and patients had similar values of age, sex, BMI, SBP, DBP, serum albumin, ALT, AST, and creatinine, as well as Child–Turcotte–Pugh scores among the groups at Week 0 and 12.

The responses of patients in terms of biochemical parameters to placebo or supplementation are shown in Table 2. All patients had similar levels of cysteine, MDA, GSH, GSSG, and enzyme activity at Week 0. The B-6 and B-6 + GSH group patients had significantly increased plasma PLP levels at Week 12 compared to the levels at Week 0. Unexpectedly, the GSH levels, and its related enzyme activities, remained steady after 12 weeks of GSH supplementation in the GSH and B-6 + GSH groups. The activity of GSH-Px significantly decreased in patients taking placebo and B-6 supplementation at Week 12. Multiple regression analyses showed that neither vitamin B-6 nor GSH supplementation had significant effects on the indicators of oxidative stress and antioxidant capacities after adjusting for potential confounders (data not shown).

Since vitamin B-6 and GSH supplementation did not significantly improve the GSH plasma levels or related antioxidant capacities, we decided to examine any changes in disease severity (Child–Turcotte–Pugh score) over time. Table 3 shows the associations of liver cirrhosis severity with biochemical parameters before (Week 0) and after (Week 12) supplementation and at the end of follow-up. The median follow-up time was 984 d (interquartile range, 835–1530.5 d; minimum, 77 d; maximum, 1689 d), and 21 patients were lost of follow-up. At the end of follow-up, the mean and standard error of the mean of the Child–Turcotte–Pugh score was 5.25 ± 0.11 for 40 patients. There were no significant associations between Child–Turcotte–Pugh score and plasma PLP, cysteine, and MDA levels at any time. Only high levels of GSH, high GSH/GSSG ratio, and high GSH-St activity at baseline (Week 0), but not at Week 12, had significant effects on low Child–Turcotte–Pugh scores at Week 0, Week12, and at the end of follow-up in all patients after adjusting for age, sex, BMI, smoking and drinking status, and follow-up time.

## 4. Discussion

Vitamin B-6 has been shown to have both direct [26,27] and indirect antioxidative effects in human body. An adequate vitamin B-6 status might shelter the patients against the high oxidative stress induced by their diseases. In our previous study, 50 mg of vitamin B-6 supplementation for 12 weeks could mediate antioxidant capacity by reducing plasma homocysteine in patients with hepatocellular carcinoma [14], in consequence, we used the same dosage (50 mg/d pyridoxine-HCl) to treat our patients in the present study. Although the plasma PLP level significantly increased 2.7–5-fold compared to the baseline level following a 12-week supplementation of daily 50 mg vitamin B-6, no apparent beneficial effects were demonstrated on oxidative stress and antioxidant capacities in patients with liver cirrhosis in the present study. Similarly, a single oral 25 mg of pyridoxine supplementation and a 28-day course of daily 25 mg pyridoxine supplementation could successfully replete plasma PLP levels in liver cirrhosis patients [17,28], but the supplementation could not improve their deranged amino acid metabolism [17]. However, it has been previously demonstrated that the administration of vitamin B-6 (100 mg/kg) considerably ameliorated the chromium-induced oxidative stress in rats [29]. Indeed, the administration of a single dose of vitamin B-6 (600 mg/kg, subcutaneous) immediately into cecal ligation and perforation-induced mid-grade septic rats could remarkably reduce acute oxidative damage in the liver [30]. Since Horowitz et al. suggested an impaired transsulfuration pathway in cirrhosis patients [31], we speculated that the antioxidant properties of vitamin B-6 might be deranged or blocked in cirrhosis, even though the plasma PLP levels increased following a large dose of vitamin B-6 supplementation.

GSH depletion could lead to the overproduction of reactive oxygen species which can disrupt mitochondrial transmembrane potential and lead to mitochondrial dysfunction [32]. As a result, the ratio of GSH/GSSG has been used to assess the status of oxidative stress in the cells [33]. Reduced GSH-Px and SOD activities of cells have been shown to prompt apoptosis [34]. Augmentation of oxidative stress has been implicated in the pathogenesis and progression of various hepatic diseases; therefore, sufficient GSH status and its related antioxidant enzyme activities are necessary to protect oxidative damage in the liver. The efficacy of GSH supplementation has been reported in various diseases, including cystic fibrosis, peripheral obstructive arterial disease, Parkinson’s disease, and autism spectrum disorders [35,36,37,38]. Different routes of administration, such as intravenous, inhaled, or oral, have been utilized. A previous study revealed that administration of oral GSH (1 g/kg body weight) significantly increased the hepatic GSH level in rats [39], and therefore, we presumed that GSH supplementation could also increase plasma GSH concentration in cirrhosis patients. Unlike vitamin B-6, the plasma GSH level and its related enzyme activities did not respond to 12-weeks of daily 500 mg GSH supplementation. An inadequate dose of GSH (500 mg/d) was considered responsible for the non-significant results in plasma level. To the best of our knowledge, this is the first study to administer oral GSH to liver cirrhosis patients; we had no reference of a safe oral GSH dose for patients with liver cirrhosis, even though a 1000 mg/d oral GSH supplement had previously been given to healthy subjects [10,11]. The recent study of oral glutathione with a dose of 300 mg per day demonstrated efficacy on the improvement of ALT levels after the four-month course of treatment for patients with non-alcoholic fatty liver disease; however, GSH levels in the plasma protein fraction decreased significantly [40]. Since we did not observe any adverse effects in cirrhosis patients during and after the intervention, a higher GSH dose could be considered. Impaired methionine degradation and transsulfuration pathway have been observed in patients with liver cirrhosis [18,41]. Indeed, it is possible that a large dose of GSH cannot compensate for the defective transsulfuration pathway in cirrhosis, which may explain why the GSH level was not significantly increased after GSH supplementation. A previous study indicated that GSH might inhibit platelet aggregation [42], and blood clotting was sensitive to the GSH redox couple in vitro [43]. INR is the assay evaluating the extrinsic and common pathway of clotting cascade to measure coagulation factors. We noticed a significant difference in INR level between vitamin B-6 and GSH groups at Week 0. Although the mean INR level was in normal range (0.8–1.2) at Week 0 and Week 12, and the significant difference in INR level between groups disappeared at Week 12, GSH metabolism might be affected by the cascade of blood clotting after GSH supplementation. In addition, the orally ingested GSH was absorbed in the blood and incorporated into the liver to maintain the GSH reservoir or to alleviate the GSH depletion from the liver under pathologic condition of cirrhosis. Therefore, we could not rule out the possibility that there might be tissue uptake of GSH; in other words, a redistribution of GSH from plasma to liver tissue during GSH supplementation may occur since liver cirrhosis patients might require more GSH and its related antioxidant enzymes to protect their damaged liver function. However, since the hepatic GSH concentration could not be determined, we could not further elucidate the speculation. Irrespective of the cause of the lack of response of plasma GSH levels to the intervention, these may explain the lack of associations between plasma GSH levels and its antioxidant enzyme activities with indicators of oxidative stress and antioxidant capacities.

An in vivo study demonstrated that, selenium-GSH-enriched probiotics could effectively increase the hepatic GSH level and the activities of GSH-Px and SOD, as well as attenuate carbon tetrachloride-induced hepatic oxidative stress and liver fibrosis in rats; this study also demonstrated that this combination probiotics had better protective effects on liver fibrosis than selenium or GSH alone [44]. Indeed, only selenium-enriched rather than the combination of selenium and GSH probiotics could also significantly increase the hepatic GSH level, and GSH-Px and SOD activities, and further suppress hepatic oxidative stress in rats [45]. Selenium is an essential substrate for GSH-Px, and an adequate selenium status might be an important key factor in GSH-related antioxidant capacities. The non-significant beneficial effect of GSH supplementation on oxidative stress and antioxidant capacities might be due to inadequate selenium status in our cirrhosis patients. Since the selenium concentration was not analyzed in our study, the importance cannot be assessed further.

The Child–Turcotte–Pugh score is a well-established classification to evaluate the severity of liver cirrhosis. Although we did not observe beneficial effects of GSH supplementation on oxidative stress or antioxidant capacities in patients with liver cirrhosis, the valuable finding of this study was that there were negative associations of the GSH level, the GSH/GSSG ratio, related antioxidant enzyme activity, and Child–Turcotte–Pugh score before, during, and after the intervention in our patients. The association between GSH status and cirrhosis severity was also observed in our previous study [46], as well as other studies [3,4,19]. Patients with liver cirrhosis appeared to increase their GSH turnover and antioxidant enzyme activity to cope with oxidative stress in concordance with the disease severity. Although we could not further demonstrate the use of oral GSH supplementation as an effective alternative therapy to counteract liver damage in cirrhosis patients, the strategy of increasing or maintaining adequate GSH-related antioxidant capacities is still needed for patients with liver cirrhosis.

The uniqueness of the study lies in the oral GSH supplementation for patients with liver cirrhosis, which has not been previously explored. In addition, we performed a follow-up (median time: 984 d) after the completeness of the intervention, where the long-term effect of vitamin B-6 and GSH supplementation on clinical outcomes could be investigated. However, since we lost 21 patients to follow-up, the decreased sample size might reduce the statistical power to not reveal a significant effect of vitamin B-6 or GSH on oxidative stress, antioxidant capacities, and clinical outcomes. The second limitation was a lack of tissue GSH levels to further discuss a possible dynamic operation of GSH biosynthesis, consumption, and turnover in liver cirrhosis during GSH supplementation. Third, the etiology of cirrhosis in our patient groups comprised viral, alcoholic, and cryptogenic origin. The different course of insults in liver damage would result in different presentations of deranged GSH status. The last limitation was using the MDA level to be an indicator of oxidative stress since the MDA level might be affected by the ongoing liver injury. However, the ratio of GSH/GSSG was used to be a secondary measure of oxidative stress to confirm the findings.

## 5. Conclusions

Although the decreased GSH and its related enzyme activity were associated with the severity of liver cirrhosis, vitamin B-6 and GSH supplementation had no significant effect on reducing oxidative stress and increasing antioxidant capacities.

## Figures and Tables

**Table 1:** Demographic and clinical characteristics of patients with liver cirrhosis.

Parameters	Placebo (*n* = 14)		B-6 (*n* = 14)		GSH (*n* = 18)		B-6 + GSH (*n* = 15)	
	Week 0	Week 12	Week 0	Week 12	Week 0	Week 12	Week 0	Week 12
Age (y)	55.0 ± 3.41		62.86 ± 2.09		62.39 ± 2.35		56.40 ± 1.84	
Sex (male/female)	10/4		9/5		14/4		14/1	
BMI (kg/m^2^)	25.70 ± 1.21	25.43 ± 1.30	25.96 ± 0.91	25.87 ± 0.79	23.71 ± 0.89	23.38 ± 0.88	25.40 ± 0.67	25.82 ± 0.60
Blood pressure (mmHg)								
Systolic	136.71 ± 6.02	126.86 ± 5.39	137.0 ± 6.04	134.29 ± 7.03	127.61 ± 4.65	124.61 ± 2.38	128.53 ± 3.29	129.33 ± 2.97
Diastolic	83.14 ± 4.44	78.43 ± 3.41	78.64 ± 3.61	78.14 ± 4.26	79.44 ± 3.10	75.50 ± 1.73	79.33 ± 2.29	78.20 ± 2.70
Serum ALT (U/L)	48.21 ± 11.67	43.36 ± 10.48	56.86 ± 17.40	42.43 ± 6.38	54.22 ± 12.75	44.78 ± 8.50	73.60 ± 19.86	59.13 ± 9.81
Serum AST (U/L)	42.07 ± 8.07	42.21 ± 8.80	60.93 ± 13.92	57.21 ± 11.20	82.33 ± 42.31	38.61 ± 2.86	59.47 ± 13.97	54.67 ± 8.80
Serum creatinine (mg/dL)	0.96 ± 0.10	0.99 ± 0.12	0.78 ± 0.04	0.94 ± 0.08 *	0.88 ± 0.03	0.93 ± 0.06	1.18 ± 0.21	1.23 ± 0.23
Serum albumin (g/dL)	4.05 ± 0.19	3.95 ± 0.18	3.94 ± 0.23	3.95 ± 0.16	4.32 ± 0.13	4.22 ± 0.12	4.09 ± 0.15	4.10 ± 0.15
Serum total bilirubin (mg/dL)	0.93 ± 0.22	1.16 ± 0.18	1.29 ± 0.18	1.15 ± 0.17	1.11 ± 0.17	0.99 ± 0.12	1.59 ± 0.58	1.31 ±0.44
INR	1.10 ± 0.04 ^a,b^	1.11 ± 0.03	1.14 ± 0.02 ^a^	1.13 ± 0.03	1.04 ^b^ ± 0.01	1.05 ± 0.02	1.12 ± 0.04 ^a,b^	1.12 ± 0.05
Child–Turcotte–Pugh scores								
A (*n*, %)	12, 85.71%	13, 92.86%	12, 85.71%	12, 85.71%	18, 100%	18, 100%	14, 93.33%	13, 86.67%
B (*n*, %)	2, 14.29%	1, 0.07%	2, 14.29%	2, 14.29%	0	0	1, 6.67%	2, 13.33%
Smoking (*n*, %)	3, 21.43%		3, 21.43%		6, 33.33%		6, 40%	
Drinking (*n*, %)	1, 7.14%		2, 14.29%		1, 5.56%		4, 26.67%	

Data are presented as mean ± standard error of mean. BMI, body mass index; ALT, alanine aminotransferase; AST, aspartate aminotransferase; INR, international normalized ratio. * Values are significantly different from Week 0 within the group, *p* < 0.05. ^a,b^ Values are significantly different among groups at Week 0, *p* < 0.05.

**Table 2:** Responses of biochemical measurements to placebo or supplementation in patients with liver cirrhosis.

Parameters	Placebo (*n* = 14)		B-6 (*n* = 14)		GSH (*n* = 18)		B-6 + GSH (*n* = 15)	
	Week 0	Week 12	Week 0	Week 12	Week 0	Week 12	Week 0	Week 12
PLP (nmol/L)	67.59 ± 10.58	107.63 ± 35.69 ^b^	55.21 ± 7.37	276.56 ± 38.50 *^,a^	131.14 ± 33.80	63.93 ± 11.33 ^b^	116.74 ± 37.06	319.87 ± 52.63 *^,a^
Cysteine (µmol/L)	202.53 ± 14.75	223.62 ± 11.10	217.24 ± 13.57	209.73 ± 10.63	208.15 ± 9.76	225.74 ± 10.21 *	189.72 ± 9.25	192.22 ± 8.07
Oxidative stress indicators								
MDA (µmol/L)	0.80 ± 0.05	0.87 ± 0.08	0.77 ± 0.04	0.80 ± 0.07	0.78 ± 0.06	0.75 ± 0.05	0.81 ± 0.03	0.87 ± 0.06
GSH/GSSG ratio	0.11 ± 0.01	0.11 ± 0.01	0.09 ± 0.01	0.10 ± 0.01	0.12 ± 0.01	0.12 ± 0.01	0.12 ± 0.01	0.10 ± 0.01 *
Antioxidant capacities								
TEAC (µmol/L)	4335.34 ± 264.35	4412.06 ± 160.40	4448.85 ± 147.24	4321.28 ± 113.79	4078.22 ± 112.37	4324.72 ± 96.95	3943.14 ± 143.68	4259.35 ± 103.17
GSH (µmol/L)	72.25 ± 6.96	79.59 ± 9.81	66.54 ± 10.01	68.62 ± 10.63	77.43 ± 4.98	80.23 ± 5.76	78.14 ± 7.81	71.52 ± 7.20
GSSG (µmol/L)	666.06 ± 18.53	694.20 ± 13.70	667.05 ± 18.33	679.30 ± 15.92	673.05 ± 19.10	691.85 ± 16.17	662.61 ± 12.46	705.29 ± 17.33 *
GSH-St (nmol/mL/min)	15.97 ± 3.60	22.20 ± 4.54	16.28 ± 2.68	26.46 ± 3.30	19.47 ± 3.42	27.00 ± 2.84	18.98 ± 2.65	25.58 ± 3.36
GSH-Px (nmol/mL/min)	199.39 ± 20.74	142.82 ± 10.77 *	211.94 ± 26.26	135.35 ± 8.06 *	189.89 ± 12.57	168.70 ± 13.17	175.40 ± 16.43	151.29 ± 14.14
GSH-Rd (nmol/mL/min)	64.91 ± 4.14	68.29 ± 5.20	70.68 ± 3.98	72.73 ± 33.03	85.02 ± 12.68	69.75 ± 4.03	80.28 ± 6.80	71.64 ± 6.76
SOD (U/mL)	8.46 ± 0.93	7.81 ± 0.87	7.17 ± 1.01	8.44 ± 0.74	7.07 ± 0.73	7.36 ± 0.71	8.24 ± 1.05	9.08 ± 0.73
Catalase (nmol/mL/min)	85.68 ± 17.27	61.16 ± 10.06 *	62.91 ± 5.76	55.79 ± 8.50	85.47 ± 13.50	56.16 ± 5.53 *	79.80 ± 8.67	71.45 ± 9.56

Data are presented as mean ± standard error of mean. PLP, pyridoxal 5′-phosphate; GSH, glutathione; GSSG, glutathione disulfide; MDA, malondialdehyde; CRP, C-reactive protein; TEAC, trolox equivalent antioxidant capacity; GSH-St, glutathione *S*-transferase; GSH-Px, glutathione peroxidase; GSH-Rd, glutathione reductase; SOD, superoxide dismutase. * Values are significantly different from the Week 0 within the group, *p* < 0.05. ^a,b^ Values are significantly different among groups at Week 12, *p* < 0.05.

**Table 3:** Multiple linear regression analysis with Child–Turcotte–Pugh score as the dependent variable in patients with liver cirrhosis at Week 0 and Week 12 after adjusting for potential confounders.

Parameters	Child–Turcotte–Pugh Score		
	Week 0 (*n* = 61) ^1^	Week 12 (*n* = 61) ^1^	End of Follow-Up (*n* = 40) ^2^
	*β* (standard error)		
PLP (nmol/L)	−0.001 (0.001)		
at Week 0		−0.0002 (0.001)	−0.001 (0.001)
at Week 12		−0.001 (0.001)	−0.001 (0.001)
Cysteine (µmol/L)	0.001 (0.003)		
at Week 0		0.002 (0.003)	0.002 (0.003)
at Week 12		0.001 (0.003)	0.002 (0.003)
Oxidative stress indicators	0.573 (0.619)		
MDA (µmol/L)			
at Week 0		0.269 (0.644)	−0.159 (0.723)
at Week 12		0.830 (0.453)	−0.070 (0.419)
GSH/GSSG ratio	−8.122 (2.514) **		
at Week 0		−9.529 (2.523) ^†^	−5.928 (2.469) *
at Week 12		−6.837 (2.145) **	−3.153 (2.473)
Antioxidant capacities	−0.0004 (0.0001) *		
TEAC (µmol/L)			
at Week 0		−0.0004 (0.0002) *	−0.001 (0.0001)
at Week 12		−0.0002 (0.0002)	−0.0004 (0.0003)
GSH (µmol/L)	−0.011 (0.004) **		
at Week 0		−0.013 (0.004) ^†^	−0.009 (0.004) *
at Week 12		−0.009 (0.003) **	−0.004 (0.004)
GSSG (µmol/L)	0.001 (0.002)		
at Week 0		0.0004 (0.002)	−0.002 (0.001)
at Week 12		−0.004 (0.002) *	−0.003 (0.002)
GSH-St (nmol/mL/min)	−0.020 (0.009) *		
at Week 0		−0.022 (0.009) *	−0.022(0.009) *
at Week 12		−0.015 (0.008)	−0.010 (0.009)
GSH-Px (nmol/mL/min)	−0.0001 (0.001)		
at Week 0		0.001 (0.002)	0.001 (0.002)
at Week 12		−0.002 (0.002)	−0.002 (0.002)
GSH-Rd (nmol/mL/min)	−0.001 (0.003)		
at Week 0		0.001 (0.003)	−0.003 (0.006)
at Week 12		0.016 (0.005) **	0.007 (0.005)
SOD (U/mL)	−0.071 (0.030) *		
at Week 0		−0.030 (0.032)	0.012 (0.032)
at Week 12		−0.008 (0.034)	0.020 (0.036)
Catalase (nmol/mL/min)	−0.006 (0.002) **		
at Week 0		−0.006 (0.002) **	−0.002 (0.002)
at Week 12		−0.008 (0.003) *	−0.007 (0.004)

*n* = 61. *β*, regression coefficient. PLP, pyridoxal 5′-phosphate; GSH, glutathione; GSSG, glutathione disulfide; MDA, malondialdehyde; TEAC, trolox equivalent antioxidant capacity; GSH-St, glutathione *S*-transferase; GSH-Px, glutathione peroxidase; GSH-Rd, glutathione reductase; SOD, superoxide dismutase. ^1^ Adjusted for age, sex, body mass index, smoking and drinking status. ^2^ Adjusted for age, sex, body mass index, smoking and drinking status, and follow-up time. * *p* < 0.05; ** *p* < 0.01; ^†^ *p* < 0.001.

## Abbreviations

The following abbreviations are used in this manuscript:

ALT	alanine aminotransferase
AST	aspartate aminotransferase
BMI	body mass index
GSH	glutathione
GSH-Px	glutathione peroxidase
GSH-Rd	glutathione reductase
GSSG	glutathione disulfide
GSH-St	glutathione *S*-Transferase
INR	international normalized ratio
MDA	malondialdehyde
PLP	pyridoxal 5′-phosphate
SOD	superoxide dismutase
TEAC	trolox equivalent antioxidant capacity
